# Community and provider perceptions and experiences of cervical cancer screening in Rural Bolivia: a qualitative study

**DOI:** 10.1186/s12905-023-02500-2

**Published:** 2023-07-05

**Authors:** Armando Basagoitia, Sahai Burrowes, Maria Teresa Solis-Soto, Genevieve MacMillan, Sarah Sullivan

**Affiliations:** 1 Salud Global, Urriolagoitia #354 Primer Piso Urriolagoitia 354, Sucre, Bolivia; 2 Touro University California Public Health Program, CEHS, 1310 Club Drive Vallejo, Vallejo, CA 94592 USA; 3 Universidad de O’Higgins, Libertador Bernando O’Higgins 611, Rancangua, O’Higgins Chile

**Keywords:** Bolivia, Cervical cancer, Screening, Pap smear, Qualitative

## Abstract

**Background:**

Despite efforts to increase cervical cancer screening access in rural Bolivia, uptake remains low. Bolivia has one of the highest cervical cancer mortality rates in the Americas. As it redoubles efforts to deliver Universal Health Care, the Bolivian government needs information on the factors constraining cervical cancer screening access and utilization, especially in rural areas.

**Methods:**

Our qualitative study explored cervical cancer screening barriers and described community and provider perceptions and experiences of care. Bolivian and US researchers analyzed data collected from eight focus groups with male and female community members (n = 80) and interviews with healthcare providers (n = 6) in four purposively selected rural communities in Hernando Siles, Bolivia. Deductive and inductive codes were used to thematically analyze data using MaxQDA software.

**Results:**

Four themes emerged from the data: lack of knowledge/misconceptions, health system inadequacy, lack of confidence in providers, and opportunities for improvement. Both men and women displayed misconceptions about the causes of cervical cancer, its consequences, the recommended screening frequency, and the means of accessing care. Providers noted community members’ lack of knowledge and low risk-perception as utilization barriers but also highlighted poor health service quality and inconsistent health education as factors. Poor healthcare quality was a significant barrier; this included poor patient-provider communication, lack of transportation to screening facilities, and severe delays in receiving test results. Providers also noted problems with provider training and physical space for screening. Community members reported low confidence in nurses to perform screening, preferring doctors and specialists. They also expressed discomfort in having male healthcare providers conduct screening. Suggestions for improvements included more intensive cervical cancer outreach to rural areas and having specialists train lower-level providers to perform screening.

**Conclusions:**

Our findings suggest that poor healthcare quality has affected screening uptake in addition to physical barriers to care. They indicate a need for initiatives to reduce reporting time for Pap test results, the incorporation of community-based HPV self-sampling into screening protocols, and the implementation of programs to improve community confidence in providers’ ability to perform screening.

## Introduction

Cervical cancer is the most common cancer among women in Bolivia. It disproportionately affects the poorest and most vulnerable and has a significant social and economic impact on families and rural communities. Bolivia has the highest age-standardized incidence rates of cervix uteri in females (36.6/100,000 females), the highest number of prevalent cases, and the second highest cervical cancer mortality rate (18/100,000 females) in the WHO Region of the Americas [1]. Cervical cancer accounts for 3.04% of deaths in Bolivian women; a larger proportion of deaths than any other cancer and more than double the deaths attributed to maternal causes, HIV, or malnutrition [2]. Cervical cancer incidence is predicted to double in the next 20 years as Bolivia’s population ages [3]. Cancers are currently responsible for approximately 20% of deaths in the country and the majority of disability adjusted life years and deaths are attributable to non-communicable diseases [2].

A main factor contributing to Bolivia’s high cervical cancer mortality rates is the difficulty in implementing reliable, high-quality screening programs [4]. Despite considerable efforts to increase primary healthcare access in Bolivia, cervical cancer screening uptake remains low. According to the last comprehensive Demographic Health Survey in 2008, only 33.3% of women aged 15–49 have ever been screened in Bolivia, with only 19.3% screened in the poorest communities and 25.2% in the rural areas [5]. These low coverage rates are far from the recommended WHO target of 70% screened to reduce cervical cancer in populations [6].

Socio-cultural and health system factors drive low access to and utilization of cervical cancer screening in Bolivia. Poverty, geographic isolation, female illiteracy, lack of education, gender inequality, and discrimination against indigenous populations have created severe disparities in health outcomes between urban and rural areas [7, 8]. These disparities are compounded by health system weaknesses that have resulted in minimal human resources for health and poor insurance coverage [7, 8].

The Bolivian Ministry of Health has made significant efforts to address these disparities and systems’ weaknesses including initiatives aimed to train hundreds of primary health care doctors to work in rural areas, and a Universal Health Care system established in 2016 to increase financial access to outreach and primary healthcare services in communities [9, 10]. Since 2014, Bolivian law has guaranteed free basic primary care health services, including free Papanicolaou cytology testing [9]. According to the most recent *National Plan for the Prevention, Control, and Follow-up of Cervical Cancer*, published in 2009, annual screening tests and treatment of pre-malignant lesions should be provided by trained health workers at all levels of the health care system [9]. National screening guidelines recommend cytology (Papanicolaou (Pap) test). New prevention and diagnosis strategies, such as vaccinating against human papillomavirus (HPV) and incorporating HPV DNA testing into screening protocols, are under evaluation for rollout across Bolivia but are not yet incorporated into national guidelines [11].

As the Bolivian government redoubles efforts to deliver Universal Health Care by creating policies to promote equity and intercultural health, expand health promotion and rural health, it will need information from rural healthcare providers and communities about the quality of existing cervical cancer screening and the factors constraining its uptake. In particular, it will require information on local barriers and facilitators to healthcare access and usage [12].

Such information is largely lacking in Bolivia, where only a handful of cervical cancer studies have been conducted in recent years. In order to contribute to addressing this gap in knowledge about the barriers people face in the prevention and diagnosis of cervical cancer in rural Bolivia, our objective was to assess community members’ perceptions and experiences of cervical cancer care. The study was coordinated with local Bolivian social and health authorities concerned about high levels of new advanced-stage cervical cancer cases in their communities.

## Materials and methods

The study used a phenomenological approach to explore community and provider perceptions of cervical cancer screening barriers and their experiences of receiving and providing care. A phenomenological approach explores participants’ lived experience to consider how these experiences can be used to understand their motivation and engagement with cervical cancer screening in rural Bolivia [13].

### The research team and reflexivity

The project was developed by a male Bolivian medical doctor and public health academic (Researcher 1) and a Bolivian female public health researcher (Researcher 2) in coordination with local public health officials. Researcher 1 led project implementation and took several trips to the study area before data collection to coordinate with local leaders, obtain permissions, and pilot test data collection and informed consent materials.

The data collection team included Researcher 1, a female Bolivian Guaraní nurse (Researcher 3), and a male Bolivian psychologist (Researcher 4). This team was assisted by an American male public health/medical student from Touro University California (Researcher 5), who acted as a notetaker and logistics coordinator for the focus groups. The Bolivian psychologist (Researcher 4) conducted interviews with healthcare providers. All data collection team members were fluent in Spanish and English, and Researcher 3 was fluent in both Spanish and Guaraní. Researcher 3 had extensive experience working with all four communities. Researchers 1 and 4 had extensive training and experience in qualitative research methods.

The data-analysis team consisted of Researchers 1, 2, and two female Touro University California public health faculty members, one with expertise in qualitative research in the reproductive health sphere (Researcher 6), the other, a fluent Spanish-speaker with expertise in Bolivian research ethics (Researcher 7), and two public health students from Touro University California who were both fluent in Spanish (Researcher 8, 9). The data analysis team also leads manuscript preparation.

### Study setting and participant selection

The research team purposively selected Hernando Siles, a rural province, in Chuquisaca Department in south-eastern Bolivia, as the study site because it had low rates of cervical cancer screening and a high incidence of advanced-stage cervical cancer diagnoses among those screened. Chuquisaca Department is more rural than the national average (51% vs. 30% rural) and is 73% indigenous [14]. There is a lack of current, sub-national health statistics on the area, but the 2009 data suggests that the Departments’ primary health coverage is roughly at the national average [15].

Within Hernando Siles province, the team worked with local health authorities to select four communities, including one Guaraní indigenous community, that represented the province’s geography, socio-demographic profile, and health infrastructure. Three of the communities were located in rural areas that only had a single primary care health center as its source of formal health care. The fourth community was in the small provincial capital of Monteagudo and contained a secondary-level hospital.

Two focus group discussions (FGDs)—one for women and one for men—were conducted in each of the four communities (n = 8 FGDs). Each group had ten participants for a total of 80 community participants. Focus group participants were selected with assistance from local health authorities. Our sampling strategy was based on our desire to learn about women’s lived experiences of having a PAP test in order to gain information that would help us understand screening barriers [13]. We sampled men because machismo has been cited as a barrier to screening in the Americas [16, 17].

Women were chosen from health center registries, with the aim of finding women who had undergone screening and achieving a balance between those over and under the age of 35. Health center registries were considered representative as these centers were the only healthcare facilities in the communities and are widely used for a variety of care needs. We expected that very few local women would be unlisted in the registries and that therefore, focus group participants reflected typical women in the communities. Women who met the initial selection criteria (having been screened) were invited to participate in the focus groups through a phone call or home visit performed by their local authorities. The male partners of the women who agreed to participate were invited to join the men’s focus groups. Community leaders who are social representatives of the communities chosen according to their local customs, visited the communities before data collection and instructed the participants who had agreed to participate in the study to meet at a specified date, time, and place to join the focus groups. The project did not track the number of community members who declined to participate in the study. Focus groups were held in local schools and a hospital meeting room in one village.

We supplemented data on the community’s perceived barriers to screening with data from six in-depth interviews with health personnel in each of the four rural communities. These interviews were conducted at the same time as the community focus groups. All healthcare providers who were involved in cervical cancer screening, diagnosis, and care were eligible for inclusion in the interviews, but often there was only one health personnel (usually a nurse) at the primary care health center who was available for interviews. Four interviews were conducted at the primary health centers and two at the secondary hospital in Monteagudo.

### Data collection

The research team developed the focus group and interview guides using the Bolivian National Cervical Cancer Plan and Guidelines to develop questions regarding the appropriate care for cervical cancer patients [18]. We incorporated standard questions on cervical cancer knowledge, attitudes, and practice from existing survey instruments and interview guides, as well as general, open-ended questions on experiences with medical services and barriers to care [19, 20]. The guides were developed in Spanish and were pilot-tested by the primary investigators (Researchers 1, 2, and 3) with the support of five local health personnel, five women and five men from the communities selected for the study during coordination and scheduling visits prior to data collection field work. As a result of the tests adjustments were made to improve the clarity and comprehensibility of the focus group guides.

The Bolivian researchers (Researchers 1 & 3) conducted the focus groups together, and the Guaraní nurse (Researcher 3) conducted the focus groups in the Guaraní indigenous community in the Guaraní language. All participants provided written informed consent prior to data collection. This informed consent process, which was conducted in the participants’ preferred language, verified understanding of the project and highlighted the voluntary nature of participation in the project in accordance with current ethical research standards. Because the level of literacy among the participants varied, facilitators read the informed consent document, explaining terms as requested for all potential participants regardless of their literacy level.

During the focus group discussions, a lead facilitator asked questions, and another research collaborator took notes and recorded conversations. At the end of the discussions, the researchers gave participants information on cervical cancer, correcting any misinformation shared in the group and answering outstanding questions. The health worker interviews were all conducted by the Bolivian psychologist on the team (Researcher 4).

The focus groups and interviews lasted approximately one hour and were audio-recorded with consent. Communal dinners were provided after the focus groups and interviews were completed to compensate for the participants’ time and transportation costs.

The research study protocol was approved by the Touro University California IRB (# PH-5517-TW) on February 15, 2017, and the Comité de Bioética de la Facultad de Medicina, Universidad Mayor de San Simon, Cochabamba - Bolivia on February 20, 2017.

### Data analysis

Project researchers transcribed audio files verbatim, and Researcher 3 translated the Guarani transcript into Spanish. The recordings, transcriptions, and Guaraní- Spanish translations were imported into MAXQDA (VERBI Software, 2019), a qualitative text management software, for data analysis [21]. Data analysis was conducted on the Spanish-language transcripts. Researchers 1, 7 and 8 translated illustrative quotes into English for the manuscript.

The research team (Researchers 1 and 9) used a combination of inductive and deductive coding to identify themes that captured the participants’ barriers and facilitators related to cervical cancer prevention and care [22]. A preliminary set of codes were developed by two researchers (Researchers 1 and 9) using the data from the first focus group of women. These codes were then discussed, refined, and agreed upon by Researchers 1, 2, and 9. Researcher 9 applied the resulting codes to analyze the remaining focus group and in-depth interview transcripts. Researchers 1 and 9 held weekly meetings to discuss coding evolution and code application. Differences in understandings of the codes and disagreements about how codes should be applied were thoroughly discussed among the two researchers until a consensus was reached. Data interpretation was conducted during data analysis.

The codes were categorized into four overarching themes. The codes, themes, and illustrative quotes were then translated from Spanish to English for manuscript development. Researchers 6 and 7 reviewed and refined these thematic codes and the selection of illustrative quotes as part of their participation in manuscript preparation. Refinement consisted of rewording themes to account for a lack of clarity in translations and removing codes and themes that were outside of the scope of the study.

## Results

Four themes emerged from the data: (1) lack of cervical cancer knowledge/misconceptions, (2) health system inadequacy, (3) lack of trust in providers, and (4) opportunities for improvement. The themes and subthemes are summarized in Table 1 below.

**Table 1:** Themes and Sub-themes

Themes	Sub-themes
Lack of cervical cancer knowledge & misconceptions	• Misconceptions about causes of cervical cancer • Inability to describe symptoms • Lack of knowledge regarding screening schedule & services
Health system inadequacy	• Difficulties accessing care • Repeated delays in receiving results • Patchy, inconsistent health outreach and education • Lack of staff and a lack of space and equipment
Lack of trust in providers	• Lack of confidence & trust in the local health personnel • Lack of confidence in male providers
Opportunities for improvement	• Improve physical access to services • Increase rigorous training of local health personnel • Improve communication & coordination within health system

Both male and female community members displayed misconceptions about the cause of cervical cancer, its consequences, recommended screening frequency, and means of accessing care. Providers noted community members’ poor knowledge and low risk-perception as utilization barriers but also highlighted poor health service quality and inconsistent health education as barriers. Poor healthcare quality was a significant barrier mentioned by respondents; this included poor patient-provider communication, lack of transportation to screening facilities, and severe delays in receiving test results. Providers also noted problems with care coordination, provider training, and physical space for screening when discussing experiences providing screening services. Community members reported low levels of confidence in the ability of nurses to perform Pap screening, preferring doctors and specialists. They also expressed discomfort in having male healthcare providers conduct screening. Suggestions for improvements included improving access to care through physical services; implementing more intensive cervical cancer outreach, conducting more rigorous training of lower-level providers to perform screening, and improving communication and coordination within the health system.

### Theme 1: lack of information/misconceptions

Both male and female community members displayed a poor understanding of the relationship between HPV infection and cervical cancer, the symptoms of cervical cancer, and recommended cervical cancer screening schedule.

Community members had misconceptions about the causes of cervical cancer. One male focus group member respondent thought “it might be passed through the blood (Men’s FGD 1)”, while others thought that it was due to a “lack of hygiene” (Women’s FGD 1) or to “a residue from a miscarriage” (Women’s FGD 1). The connection between cervical cancer and HPV infection was rarely mentioned.

Cervical cancer is a tumor that appears in the uterus of women…because they have a lot of infection or [because of] not doing a Pap screening (Women’s FGD 1)

... they hit, hurt themselves, and that damage causes cancer to the cervix (Men’s FGD 4),

I think it [cervical cancer] comes when they have a home birth in the rural areas, and they don’t have all the [proper] care they have at the hospital (Men’s FGD 2)

Women always have to have at least three children because if they don’t... then they don’t get to eliminate all the bad things they have in their body... they get cervical cancer (Women’s FGD 2)

Women knew that cervical cancer was a serious and potentially deadly disease that affects the cervix but could not describe its symptoms. For example, women stated conditions such as “numbness of the feet” (Woman’s FGD 3) as symptoms. Male focus group respondents had similar knowledge gaps regarding symptoms and, in addition, low overall awareness of cervical cancer. Several male community members, such as this respondent, were unaware of cervical cancer prior to the study:

“I’ve never heard [about cervical cancer]. [This is] the first time I’m hearing about this” (Men’s FGD 4)

In interviews, providers stated that low cervical cancer awareness was an important barrier to screening. In addition, community members and providers noted a lack of information about how and when to access proper cervical cancer screening and care services. Responses indicated that community members did not know when and how often women should be screened for cervical cancer. In addition, providers noted that women sometimes think a single screening was sufficient.

I don’t know until what age it [the Pap test] should be (Women’s FGD 1 )

[Regarding the age at which women should begin to have Pap tests] It depends on the ... doctor to come and say, right? When, what year is it to be done? Because … let’s say … we as parents or as a couple can’t tell them, right? Because mostly they say it is ten years old. I’ve heard before about fifteen years old when her first [menstruation] starts. (Men’s FGD 1)

They think that “once it [the Pap test] is done, I’ve done it,” and that is it for their whole life (Provider Interview #5)

Male community members also held misconceptions about the availability or accessibility of screening and treatment services. Some, like this community member, thought that screening was only offered at certain times of day at certain times of the year.

That [screening] is not all the time. There are seasons. It can’t be done any day (Men’s FGD 3)

Others did not realize that the Pap exam was free.

‘Of course the exam costs ... I don’t know how much it costs, but of course, it costs … and if there is an infection, I think the treatment costs too (Men’s FGD 3)

The health personnel interviewed mentioned that communities do not understand the severity of cervical cancer nor the reason or required schedule for Pap tests, especially among teenagers. They attributed this lack of knowledge in part to poor health education outreach on their part and low participation in existing health outreach events.

“Some take it [cervical cancer] with ... some responsibility, right? But for others it is, it is as if they were told you have the flu, and they do not give it much importance because I don’t know, there is no consciousness yet, right? (Provider Interview 1)

“That’s the big problem, isn’t it? We lack an [health] education among people … especially adolescents, the problem of HPV awareness, for example, is serious in ... in adolescents, right?” (Provider Interview 4)

“Generally, men don’t attend [community health meetings]. It’s necessary to insist… with great insistence, they attend. They attend community meetings, but [for community meetings on] health they do not appear” (Provider Interview 2)

### Theme 2: health system inadequacy

The second broad theme to emerge in interviews and focus groups was the inadequacy of current cervical cancer screening services. Poor physical access to health education and screening services and delays in receiving feedback after screening were mentioned barriers to cervical cancer screening and diagnosis. Women’s focus group participants discussed the difficulties of accessing care in rural communities that lacked comprehensive medical facilities and laboratories. They acknowledged that there were health centers located near their communities but stated that they still needed to walk for several hours to access these facilities, often in harsh weather, crossing rivers, and other rough terrains. They bemoaned the lack of public transportation to these health centers.

Participants noted that the lack of on-site laboratories was particularly concerning because Pap test samples obtained in rural areas needed to be sent to laboratories located far away, and this caused severe delays in receiving results. In addition, the women stated that health personnel often did not provide them with a follow-up date to obtain their Pap test results, but rather that there was an understood waiting period for the communities of approximately two to three months for results to return. As a consequence, the responsibility fell on the women to frequently travel back to health centers to obtain their results. As one woman noted, “we have to come [continuously] to ask…” [for the result] (Women’s FGD 1 ). Women complained that they never received their Pap test results and believed that the results must have been misplaced or lost. They noted that they rarely received an explanation for the delays in receiving results. One woman noted that for her Pap test results, “it has been one year … they don’t give them, they always get lost, they say” (Women’s FGD 1). Men also noted problems with obtaining results.

I don’t know [about my partner’s Pap test results]; she hasn’t told me ... because she hasn’t brought me the results since a year ago. There are no results, and we do not know if she is sick or healthy….(Men’s FGD 3)

The repeated delays in receiving results had soured community members on cervical cancer screening, with numerous respondents in both the male and female focus groups saying that they no longer saw the point in continuing screening. According to guidelines, clients should receive Pap test results in writing from the facility at which they were screened. However, in practice, results are usually delivered through oral communication with the provider. Clients travel to the facility to get this information, often without knowing ahead of time whether their results are available.

When I had it done, they didn’t give me a remedy or result, and since then, I don’t want to go get a Pap smear because they didn’t give me the answer. (Women’s FGD 2)

They don’t give us [the results], so that’s why we don’t want to; we don’t want our wife to get it [screening] because they never give us the results. (Men’s FGD 4)

Providers were aware of delays in the communication of Pap test results and noted that this reduced demand for screening, with one provider stating that because of delays, “people hardly want” Pap tests (Provider Interview 5). In addition, they report that laboratory staff often complained that the samples were inadequate, which made analysis difficult and affected the quality of the results and diagnoses. One provider stated that “[Laboratory staff say that:] they [health personnel] don’t handle it [Pap test samples] well, we [health personnel] get samples handled poorly… (Provider Interview 6). Even when Pap test results are shared, community members, particularly men, said that they sometimes did not understand the results and wanted better communication on the meaning of Pap tests and their results.

That too, we want them to explain the analysis results and the results they give. So that we can also explain to our wives and tell them to have the Pap test (Men’s FGD 4)

Geographic inaccessibility made contacting communities difficult and resulted in patchy, inconsistent health outreach and education activities in rural communities. This lack of outreach by health professionals was mentioned by community members, particularly men, who noted the lack of visits by more senior health professionals and a lack of long-term engagement and one-on-one dialog on the part of health educators.

No, … but the doctor has never been asked to come to the meeting to talk to us, to explain what diseases are like, nothing (Men’s FGD 4)

They [health educators] come, weigh the children, measure them, give them tablets, and that’s all… bye, go home to heal, they say. And they don’t explain anything to us. They visit nobody. Why are we going to lie? They have never come to the house, and we would like them to come and explain to us (Men’s FGD 3)

But, in addition to the physical difficulties in reaching communities for health education, providers noted that the health educators were often not qualified and that there was conflicting health information being provided by the different organizations providing cervical cancer screening information in the community.

Health information is also given, but sometimes they bring a student from ... an intern, for example, or a general practitioner who is on duty. They who do not have all the knowledge, let’s say, to give the information” (Provider Interview 3)

But the big problem is that, the [cervical cancer] information [NGOS provide] is not similar to all” (Provider Interview 1)

Other health system barriers to cervical cancer care that emerged from the interviews with providers included a lack of staff and a lack of space and equipment for provider training, especially training to collect Pap test samples.

It [lack of staff] is a problem, for years we asked [for more staff], according to the rules, for example, there should be … a regular nurse and an auxiliary for each shift. It is not fulfilled; we do not have this. We have a nurse who is our auxiliary or one who is in two services, right?… the clinics, none of the clinics has a single nurse (Provider Interview 6)

We requested [in the past] any training, and it was given to us. …Now, there is absolutely no training, and, as I said, we need to read, and update ourselves, all those things [regarding sample collection], right? (Provider Interview 1)

Poor service quality was laid at the feet of the health authorities, who were said to lack strategy and robust plans. Providers felt that authorities only cared about the Pap test coverage numbers rather than the quality of care or funding for diagnosis and treatment.

Everything is coverage, coverage and they [health authorities] say, “Come on!” they just want to see how many [women with PAP test] we have, how many we are doing, etc. (Provider Interview 1)

### Theme 3: lack of confidence in mid-level and male providers

Related to the theme of health system weakness, a separate and consistent theme that emerged in the focus groups was an underlying lack of confidence and trust in the local health personnel. Community members did not feel that the health personnel met their health needs or expectations of quality care. As noted in the health system inadequacy theme, community members were frustrated by delays in getting Pap test results and poor communication with the health personnel in their primary health care centers. However, in addition to this, there was a seemingly deeply held belief that local health personnel at these centers were not skilled enough to collect adequate Pap test specimens. Both men and women in the community stated that they trusted gynecological specialists, especially female gynecologists, and preferred them to local health providers for performing Pap tests. Distrust of local providers and a questioning of their skills were reinforced by past negative experiences with the health system

[Discussing why women do not have Pap tests at the local health center] “it is that the doctor is a specialist … and here it’s only a nurse” (Women’s FGD 1)

I think that a gynecologist specialist knows what part [of the cervix exactly they are going to take samples [from] … a general doctor is not as often in that zone [of the body] and maybe can miss” (Women’s FGD 3)

Even if they [nurses] have been trained, there will always be mistrust. The work they do will never be good. If there is doubt, it [screening] is not going to happen even if there is the opportunity” (Men’s FGD 2)

Yes, it [the test] has to be with a doctor because the nurse does not know how to do the exam” (Men’s FGD 3).

Related to the lack of trust in local providers was a lack of confidence in male providers to perform Pap tests and a strong preference for female providers citing comfort, relatability, and trust as reasons for this preference

Yes, well, for us, a woman [gives us] more confidence” (Women’s FGD 1)

Why [do I prefer female providers]? because between women, there is more trust…I would ask her for advice, and, as a woman, she would know (Women’s FGD 1)

[Pap tests should be] with women always; I imagine they [female users] have more trust in women. Especially the gynecologist. If it’s a man, some [women] don’t want to [get tested]. If there are only men, women don’t want [screening]. (Men’s FGD 3)

“It has to be a doctor. A woman should assist because there is more trust because among women they have no shame. But if the doctor does the test… mmmm I think they [women] are ashamed” (Men’s FGD 1)

Although healthcare providers noted that machismo had decreased as a barrier to men supporting their female partners to be screened, they stated that fear and shame persist and that female doctors are preferred for screening.

That [machismo] has been decreasing. It is not like it was before; there is no such jealousy. I mean, we tell the couple, and now, well, it is ... it is ... it is Ok for them (Provider Interview 3)

In interviews, providers also noted that poor quality of care had reduced trust in providers. Most communities trusted traditional healers more than formal care providers, seeking care from these healers before getting formal care.

First [point of contact] is the healer and according what he tells them, then they come [to the health service], when he tells them “this isn’t for me, it’s for the medic .” He even says which doctor [to visit] “Go to sees this doctor” (Provider Interview 1)

### Theme 4: opportunities for improvement

All focus groups and interviews contained recommendations for improving cervical cancer prevention and care services and for increasing access to reproductive health care in general. However, suggestions for change differed markedly between the three groups of respondents.

Women’s suggestions centered on improving physical access to services. For example, several women in focus groups suggested extending public transportation schedules to facilitate travel to and from health centers. Male community members’ suggestions focused on improving and increasing the cervical cancer health education they received so that they could better support their partners. They requested more frequent community health education talks and, overall, expressed a desire for more leadership and coordination on the part of health professionals in health education efforts.

It would be good if it [health education] would be organized from above, right? Because we don’t know when they come, when they will do it so, I think the doctors should communicate: “Well, people, this is going to be done.” It is necessary for the doctor to set a date to hold the meeting, another date to test all the women, so everyone gets ready (Men’s FGD 4)

If it there is a talk and it turns out that not a single woman appears, then all husbands should participate, and to generate more confidence, we should go with our partners, take them. If she is a little embarrassed, if she is a little shy, the husband should be sitting there to accompany her for the doctor to explain (Men’s FGD 2)

Following from the theme of distrust in mid-level providers, we found that community members recommended more rigorous training of local health personnel on properly performing Pap tests, preferably by gynecological specialists.

A bit more training [by specialists lasting] about two months and [community center health personnel] should be ready (Women’s FGD 1)

In contrast to community members, health personnel’s suggestions focused less on improving access to services and more on improving communication and coordination within the health system to speed up the delivery of test results and expand the scope of services. For example, several providers noted the need for better communication with NGOs, family community doctors, and traditional healers. Specifically, they noted that these actors might have existing relationships with communities that the formal health sector lacks and that they could build on to extend their reach.

What is good about CIES [an NGO], is that it moves [around to] all people in the countryside,… [and] captures patients there (Provider Interview 1)

Providers also noted recent changes in health recordkeeping and health registration that might facilitate patient follow-up and promote screening and recommended their expansion. The increasing inclusion of mobile phone numbers in health center registries was mentioned, which allowed easier communication of Pap test results.

We have the patient’s phone numbers, so we [can let patients] know as soon as the results have arrived. Now that [the results] are coming out quickly, in a week they will be ready (Provider Interview 1)

Yet another strategy that we have taken is [communicating with patients]…by mobile phone (Provider Interview 2)

## Discussion

This study explored community and provider perceptions of cervical cancer screening barriers and their experiences of care. Four themes emerged from focus groups and interviews with providers: (1) poor information/misconceptions regarding cervical cancer, (2) health system inadequacy, (3) lack of confidence in providers, and (4) opportunities for improvement. These themes were consistent across all four communities. Women had solid awareness of cervical cancer and the need for screening but had difficulty accessing care, were distrustful of health center staff, and were frustrated in their past experiences with screening, in particular, delays in receiving test results. Their male partners had low cervical cancer awareness and harbored many misconceptions about the causes of cervical cancer and its symptoms. They were equally frustrated with the patchiness of health education and the lack of feedback regarding test results. Neither group had a solid knowledge of recommended screening frequencies. Both men and women questioned the quality of care that could be provided by local health center staff and preferred specialists. Providers also mentioned low cervical cancer knowledge, poor physical access to services, delays in receiving results, and barriers to screening, but they were primarily concerned with a lack of resources to deliver care and poor coordination within the healthcare system and between the public health system and non-governmental service providers.

### Low knowledge and many misconceptions

The finding of patchy cervical cancer knowledge is in keeping with recent Bolivian studies that found that cervical cancer knowledge is low even among highly educated women [11, 23]. The emergence of this theme suggests that there is significant room for improvement in the health outreach and screening promotion activities currently being implemented in rural areas. Improving knowledge about cervical cancer and screening services is important because studies across a range of low- and middle-income countries and in Bolivia itself suggest that knowledge is a crucial factor in screening uptake [24–28]. The need for more intensive health education may be particularly acute for men because their misconceptions seem to be more pronounced than women’s and because they seemed eager to support their partners in attending health information sessions and screening services.

### Distrust of midlevel & male providers

The lack of understanding regarding provider roles is closely related to our second theme, a distrust in midlevel providers and male providers to perform screening services. It is not clear from our results whether the distrust of midlevel providers was warranted due to low competence or whether it was primarily an artifact of our respondents’ understanding of what “quality” care entailed. Given the stated frustration with the weak performance of their local health center staff, poor quality of past care is a reasonable explanation for the preference for specialists. Our findings of distrust in midlevel providers and screening services, in general, are supported by studies that have found that distrust in health systems in low- and middle-income countries is common [29].

The preference for female providers for cervical cancer screening is also common and has been reported elsewhere in Bolivia and neighboring countries [23, 30, 31]. While there was a strong preference for female providers and feelings of shame and embarrassment about having male providers performing screening, the intense stigma surrounding cervical cancer, which is a common barrier to screening in other low-income settings and neighboring countries, was not a commonly mentioned barrier here [27, 30, 32].

### Health system weakness

While many individual-level barriers to addressing cervical cancer remain present in Bolivia, our findings highlight the fact that the country’s health system barriers—operations, personnel, and material resources—also remain issues of great concern and may reflect a growing recognition of how deeply systematic factors influence perceptions of the quality of cervical cancer prevention and care activities in the country.

Our most clearly and consistently articulated theme was that poor cervical cancer screening quality reduced demand for services. Poor quality of care—delays in obtaining results, unclear patient-provider communication, lack of consistent health outreach, and poor physical access to care—seemed to lead directly to low demand for screening and overall distrust in the health system. Our respondent’s frustration with delay is well-founded, as studies in Bolivia have found that more than half of women screened using Pap smears were lost to follow-up, primarily because of delays in obtaining results [33].

These delays ​​ could be related to deficient sample collection or handling which was mentioned by our study’s respondents. Bolivia’s current guidelines do not establish exclusive roles for sample collection, so the collection and handling of samples can be carried out by any health personnel at health posts in rural areas. Sample analysis occurs in a variety of locations (e.g., in a nearby municipality or further away) depending on workload and the availability of qualified personnel. These facilities have varying degrees of oversight and technological capacity and there is wide variation in the number of intermediaries involved in handling samples with few controls to ensure that samples are handled adequately. In addition to the poor quality of sample collection and handling, studies in other countries suggest that workload backlogs and the lack of technology and information systems and oversight mechanisms may also contribute to delays in diagnosis [34, 35].

Quality of patient-provider interactions has been shown to influence trust in the healthcare system and, in return, satisfaction with services [29, 36, 37]. Studies in high-, low-, and middle-income countries have found that structural barriers, poor communication, confidentiality breaches, and delays in providing results reduce screening uptake [30, 38, 39]. In neighboring Peru, a study found that prior experience with test results, delay, and a history of mistreatment by health personnel was negatively associated with screening uptake [30]. Like our study, one of the few other recent examinations of barriers to cervical cancer screening in Bolivia, conducted in Cochabamba city, also found that factors such as wait times and poor communication with providers were more commonly cited barriers to care compared to cost. As in our study, they also found that fear of the screening procedure, language and cultural barriers, and lack of spousal support were not frequently mentioned barriers to screening [23]. In rural Guatemala, distrust of the health system and long-turnaround times for screening results were associated with loss to follow-up post-screening [40]. Reviews of studies on screening barriers and facilitators have found that good relationships with providers facilitate screening among the young [26].

### Clinical and policy implications

Our findings suggest that the Bolivian Ministry of Health and other service providers should consider pivoting towards promoting quality of care rather than focusing intensely on increasing coverage numbers. While poor geographic access to health centers remains a barrier, and one mentioned by the women undergoing screening in our study, quality of care at these centers seems equally important for increasing service utilization. The observed preference for specialists and distrust of midlevel providers suggests that refresher courses and increased supervision and support for rural providers could complement intensified health education/outreach to build trust in providers. It is increasingly acknowledged that enforcing competence levels for providers and paying attention to user experience is foundational to health system strengthening and that user experience, and system confidence, are significant “blind spots” in health research [29].

The lack of trust in providers and seeming greater trust that community members place in traditional healers point to the potential of incorporating traditional healers into cervical cancer screening initiatives. Because there is significant regional variation in the strength of relationships with traditional healers, this strategy might have to vary by region. In addition numerous studies have shown that the integration of traditional healers into biomedical practice and health promotion is challenging and complex due to substantial training needs of healers, the lack of systems for referral between traditional and biomedical services, and the discrimination and hostility of biomedical staff towards traditional healers [41]. In the Bolivian context, our findings also suggest that a lack of consistent outreach and interaction with the health system is at the root of distrust. Any programs to integrate traditional healers must be conducted in conjunction with more rigorous outreach initiatives. Nevertheless, traditional healers remain an untapped and potentially powerful ally in comprehensively addressing cervical cancer and wider population health in rural communities and there is some evidence for their effectiveness in health promotion [42, 43].

Overall, our findings suggest that more sustained and consistent health outreach activities are required in these rural communities. The need for more outreach and engagement is underscored by the findings of poor knowledge and common misconceptions about cervical cancer. Any future cervical cancer screening education in Bolivia should clarify how often screening should be done and when and where it is available, as this was a knowledge gap in our study. Health educators may also have to educate people about the screening process and what kind of healthcare providers are appropriate for carrying out screening, to counter community views of screening as something that is technical and complex, requiring specialized care.

Together, the problems of geographic access, under-resourced facilities, low trust in the health system, and a stated desire for more sustained health education/engagement in rural communities suggest that community-based HPV self-sampling screening approaches may be appropriate in these rural communities. It is surprising that providers in our study did not suggest changing the screening procedures to remove the need for laboratory diagnosis using microscopes. Provider recommendations for improving care centered on expanding the geographic scope of existing screening services, providing more human and financial resources, and improving communication within the system rather than employing new screening strategies. This could be due to a pragmatic understanding that health system resources are scarce and the system’s capacity to implement significant changes is limited, given its constraints. However, given the depth of negative experiences with delayed Pap results and the seemingly chronic problems with understaffed labs, our findings lend support to others who suggest that the country should move away from Pap tests as primary screening tools towards HPV DNA testing and self-collection of samples when possible [11]. Any revised cervical cancer screening policies and strategies must include proper health promotion, training, and support as well as quality control measures to ensure the successful roll out of HPV DNA testing in Bolivia. Health education campaigns would be needed to address women’s lack of confidence related to self-collection and promote the HPV self-collection approach as easy, comfortable and without pain [11]. Such approaches have been shown to be acceptable across a range of rural low-income country settings and among Bolivian women [4, 11, 44–47]. However, even with self-sampling, providing screening services will also require knowledge and decision-making skills to interpret and act on the results.

### Limitations

The following limitations should be considered when interpreting the findings of this study. First, there was a marked difference in the richness and detail between the male and female focus groups. The main facilitator for three of our women’s focus groups was male, and the women with whom he spoke may have been uncomfortable speaking to him about their reproductive care. In addition, the cultural and educational differences between facilitators and respondents may have limited our ability to engage fully with participants, translate abstract concepts, and draw out detailed responses. These contextual factors may have affected the richness of the language participants used, which is the basis of our study results. The study is also limited by the inability to explore variation in themes by socio-demographic characteristics because this descriptive and demographic data on participants was not collected.

Our third major limitation is that while transcripts were coded in Spanish, the condensing and interpretation of codes were conducted using English translations that may have flattened the nuances of the respondents’ speech. Finally, this was a small study in one geographic area and may not reflect the views of other rural communities in the country. We intended to explore this phenomenon in a population that experiences this situation intensely. Therefore, representativeness was not our goal.

Despite these limitations, this study makes a meaningful contribution to the understanding of cervical cancer screening utilization in Bolivia as there is little research on cervical cancer screening perceptions in Bolivia, and ours is one of only a handful of qualitative examinations of existing (rather than pilot) screening services. Further research on Bolivian women’s experiences and perception of screening is needed to explore our “missing” themes around stigma and to study in more depth, embarrassment, and fear of cervical cancer screening procedures to confirm their role as barriers to care in the country.

## Conclusions

Our findings suggest that in addition to physical barriers to care, past experiences of poor-quality screening care and the resulting distrust in the healthcare system may be a significant barrier to cervical cancer screening in Bolivia. They indicate a need for intensive initiatives to reduce reporting time for Pap test results and programs to improve community confidence in providers’ ability to perform screening.

## Data Availability

The datasets generated and analyzed during the current study are not publicly available due to the constraints of our ethical review but are available from the corresponding author on reasonable request.

## Abbreviations

DNA: Deoxyribonucleic acid
FGD: Focus group discussions
HPV: Human papillomavirus
Pap: Papanicolaou
